# Chemical Diversity of UK-Grown Tea Explored Using Metabolomics and Machine Learning

**DOI:** 10.3390/metabo15010052

**Published:** 2025-01-15

**Authors:** Amanda J. Lloyd, Alina Warren-Walker, Jasen Finch, Jo Harper, Kathryn Bennet, Alison Watson, Laura Lyons, Pilar Martinez Martin, Thomas Wilson, Manfred Beckmann

**Affiliations:** 1 Department of Life Sciences, Aberystwyth University, Aberystwyth SY23 3DA, UK; jsf9@aber.ac.uk (J.F.); aan@aber.ac.uk (A.W.); lal8@aber.ac.uk (L.L.); mam167@aber.ac.uk (P.M.M.); tpw2@aber.ac.uk (T.W.); meb@aber.ac.uk (M.B.); 2 Dartmoor Estate Tea, Furzeleigh Farm, Ashburton, Newton Abbot TQ13 7JL, UK; jo.harper4@gmail.com (J.H.); dartmoorestatetea@gmail.com (K.B.)

**Keywords:** *Camellia sinensis* L., Flow Infusion Electrospray Ionisation Mass Spectrometry (FIE-MS), metabolomics, random forest classification, cultivars, geographical location, temporal factors

## Abstract

Background/Objectives: Dartmoor Estate Tea plantation in Devon, UK, is renowned for its unique microclimate and varied soil conditions, which contribute to the distinctive flavours and chemical profiles of tea. The chemical diversity of fresh leaf samples from various garden locations was explored within the plantation. Methods: Fresh leaf, which differed by location, cultivar, time of day, and variety, was analysed using Flow Infusion Electrospray Ionisation Mass Spectrometry (FIE-MS). Results: Random forest classification revealed no significant differences between Georgian N2 cultivar garden locations. However, a significant degree of variability was observed within four tri-clonal variants (Tocklai Variety) with TV9 exhibiting greater similarity to the Georgian N2 cultivar compared to TV8 and TV11, while TV11 was found to be most like TV1. The intraclass variability in leaf composition was similar between the varieties. We explored the metabolic changes over the day in one variant (*Camellia assamica* Masters), yielding a model with a significant R^2^ value of 0.617 (*p* < 0.01, 3000 permutations). Starch and sucrose metabolism was found to be significant where the abundance of these chemical features increased throughout the day and then began to decrease at night. Conclusions: This research highlights the complex interplay of cultivars, geographical location, and temporal factors on the chemical composition of tea. It provides insightful data on the metabolic pathways influencing tea cultivation and production and underscores the importance of these variables in determining the final chemical profile and organoleptic characteristics of tea products.

## 1. Introduction

Tea is made from the leaves of the plant *Camellia sinensis* L. and is a widely consumed UK beverage, with a rich cultural and agricultural heritage spanning thousands of years [1]. The scientific exploration of tea has been significantly advanced by contributions from China and India, two of the largest tea-producing nations.

The regular consumption of tea promotes wellness, in particular, improved ageing, and a reduction of cardiovascular diseases, cancers, hepatopathy, obesity, and diabetes mellitus [2,3]. These benefits are largely attributed to the diverse bioactive compounds present in tea, which have been the subject of extensive research. Tea contains an array of primary and secondary metabolites, including sugars, polyphenols, amino acids, alkaloids, and volatile organic compounds, which contribute significantly to its health-promoting properties [2,3]. Studies have highlighted the role of plucking season, shading, and soil conditions, cultivar development, and the impact of climatic variations on secondary metabolites, which contribute to tea’s metabolomic profile [4,5,6,7]. The importance of genetic diversity, environmental influences, and targeted breeding objectives has been demonstrated in understanding and enhancing the chemical composition and quality of tea [8]. These variables not only affect the chemical profile of tea but also its sensory attributes, including flavour, aroma, and colour, and health-promoting attributes, underscoring the complexity of tea as a functional food.

Advances in tea research have provided critical insights into the biosynthesis of bioactive compounds such as polyphenols, amino acids, and alkaloids. Studies have emphasised the impact of traditional practices, such as fermentation and processing, on tea quality, authenticity, and flavour [9,10,11].

Metabolomics has emerged as a pivotal tool for evaluating the chemical composition, quality, and authenticity of tea and tea products. An advanced, non-targeted metabolomics approach facilitates the unbiased screening of primary and secondary chemical compounds [12], allowing researchers to investigate subtle variations between tea samples based on their origin, processing methods, and storage conditions, alongside multiple other factors. Studies have demonstrated the effectiveness of metabolomics in distinguishing tea varieties and assessing quality markers [13,14,15]. Additionally, metabolomic approaches have been used to investigate the age-related differences of tea leaf metabolites in the fresh leaves collected from tea plants aged 8 and 25 years [16]. By combining metabolomics with traditional sensory evaluations and modern analytical techniques, a comprehensive understanding of tea’s complexity and health benefits can be achieved.

Modern analytical technologies, such as high-resolution metabolomics, provide insights into plant metabolism and the biosynthesis of secondary metabolites [17]. These methods allow the exploration of the complex mechanisms of plant growth, development, and responses to environmental stimuli, thereby advancing our understanding of plant chemistry. Flow Infusion Electrospray Ionisation Mass Spectrometry (FIE-MS) can be used to produce a non-targeted comprehensive overview of the chemical content of biologically derived material as a high throughput metabolite fingerprinting tool [18]. Data obtained can be analysed using spectral binning [19] and further down-stream analysis. Machine learning using computational algorithms that can learn patterns, relationships, and insights from metabolomic data and create hypotheses. Hydrophilic Interaction Liquid Chromatography (HILIC) has the ability to separate polar compounds, such as sugars, amino acids, and organic acids, which are key metabolites in tea. This technique complements the global fingerprinting provided by FIE-MS by enabling confirmation of specific metabolites through retention time matching and MS/MS analysis.

Tea represents a cultural heritage but is also a functional beverage with significant health-promoting properties. Ongoing research into the varying bioactive compounds of tea continues to shed light on its multifaceted benefits, ensuring its enduring relevance in both dietary and therapeutic contexts. These findings provide a broader context for understanding the unique characteristics of tea grown in non-traditional regions such as the UK, which is the focus of this study.

This current study leverages advanced analytical techniques and machine learning to investigate the chemical diversity of tea grown in the UK. The Dartmoor Estate Tea plantation, located in Devon, UK, is known for its unique microclimate and soil diversity, which provide an unconventional setting for tea cultivation. This study focuses on six tea varieties grown at the estate, including the Georgian N2 cultivar (GRGN) and four Tocklai Tri-clonal variants (TV08, TV09, TV11, TV01), as well as *Camellia assamica* (*Sin.Ass*). These varieties were selected for their adaptability to diverse environmental conditions and their potential for chemical diversity.

## 2. Materials and Methods

### 2.1. Tea Plants

Six tea varieties were available at Dartmoor Estate Tea, UK, in September: GRGN (ex-Soviet Georgia commercial seed; 535 plants available and 48 plants were randomly sampled between two gardens); TV 08, 09, 11, and 01 (*Camellia sinensis* originally sourced from the Tocklai biclonal seed series TS506, TS557, TS589, and Tocklai Darjeeling commercial seed variety BB668, respectively; 860 available plants and 128 plants were randomly sampled between two gardens); and Sin.Ass, which was Tocklai Biclonal TS378 (1560 available plants in one garden; 64 plants were randomly sampled). A randomised sampling map was computer-generated before the investigation to account for batches, location (garden/plot), and time taken (Supplementary Data, Figure S1). All seeds were supplied by Teacraft Ltd., UK. These seed-derived varieties were previously misattributed as Tocklai TV vegetatively propagated clones (TV8, TV9, TV11, and TV1) in the earlier manuscript. However, we now clarify that these are not vegetatively propagated clones but seed-derived lines, and the Teacraft Ltd. codes (TCL01, TCL08, TCL09, TCL11, and TEV01, respectively) should be used throughout this revised version to reflect their correct provenance (TS378, TS506, TS557, TS589, and BB668, respectively). The Georgian seed (GRGN) was collected from abandoned tea estates in western Georgia, representing a mixed genetic background rather than a specific cultivar [20].

### 2.2. Fresh Leaf Sample Collection

For metabolome samples we took six laminar leaf punches using a single hole punch from three plants (biological replicates) for each experimental point, selecting the top two youngest leaves ensuring standardisation. These punches avoided any major leaf veins. We placed the leaf punches into a 2 mL Eppendorf tube containing a 5 mm diameter steel ball bearing using tweezers and then immediately placed the Eppendorf tubes into a Dewar to snap freeze in liquid nitrogen. We cleaned the single hole punch after each use by spraying with 70% ethanol.

For temporal sampling, the same method was applied, and samples were collected at 09:00, 12:00, 18:00, and 00:00, of the *Sin.Ass* variety, over one single day.

All leaf disc samples were frozen in liquid nitrogen immediately when they were collected in the field, then transported in liquid nitrogen and then stored at −80 °C.

### 2.3. Sample Preparation and Extraction

All procedures were carried out on ice. Mixer mill sample holders were pre-chilled at −80 °C. Bligh and Dyer extraction mix (chloroform:methanol:water 2:5:2) was prepared in advance and chilled to −20 °C, then stored on ice during use.

It was vitally important not to allow the samples to defrost during milling, so all sample tubes were first placed in liquid nitrogen before transferring to pre-chilled mixer mill holders. The samples were milled for 30 s at 30 Hz then placed in liquid nitrogen once more. If necessary, the milling process was repeated until a fine powder was obtained. The ground leaf material was immediately extracted by adding 1 mL of Bligh and Dyer extraction mix to each tube. The weight of milled material in each tube was not recorded, as a degree of uniformity was expected because each tube contained six leaf discs of uniform size. All samples were vortexed then shaken at 4 °C for 20 min before centrifugation at 13,000× *g* and 4 °C for 5 min. The supernatants were transferred into new labelled Eppendorf tubes and stored at −80 °C prior to analysis.

### 2.4. Sample Preparation for Analysis

The tea leaf extracts were defrosted, vortexed, and spun down (13,000× *g* and 4 °C for 5 min) before aliquoting. A preliminary trial had demonstrated that the optimum dilution for the samples was 1:10, so dilutions were prepared using Bligh and Dyer extraction mix before aliquoting 100 µls into HPLC vials with 200 µL inserts. For each sequence run, a quality control sample was prepared by combining 20 µL aliquots from all diluted samples in the run in a separate 5 mL Eppendorf tube and mixing before transferring 200 µL into an HPLC vial. Bligh and Dyer extraction mix (200 µL) was used as the control.

### 2.5. Flow Infusion Electrospray Ionisation Mass Spectrometry (FIE-MS)

FIE-MS was performed using an Exploris 120 mass analyser equipped with a Dionex Vanquish UHPLC system (Thermo Scientific, Waltham, MA, USA). Metabolite fingerprints were generated in both positive and negative ionisation modes, in a single run.

All samples were randomised to minimise batch effects using a computer-generated randomisation sequence. This ensured that samples from different locations, cultivars, and time points were evenly distributed across analytical runs. Samples (20 µL) were injected into a flow of 100 µL min^−1^ methanol:water (70:30, *v*/*v*). Quality control (QC) samples were prepared by pooling aliquots from all sample extracts, and these were injected at regular intervals during each sequence to monitor instrument performance. Ion intensities were acquired between *m*/*z* 55 and 1200 for 3.5 min at a resolution setting of 120,000, resulting in 3 (±1) ppm mass accuracy. Tuning and ESI source parameters were set according to the manufacturer’s recommendations. Following data acquisition, Chromeleon.cmbx files were first exported to .raw files and then converted to the .mzML open file format and centroided [21] using msconvert (TransProteomicPipeline) [22]. Spectral binning was applied using the R package binneR [19] and then standard post-acquisition processing routines were applied, including occupancy and QC filtering (Figure S2). Data were normalised and log2 transformed. Putative molecular formulas were generated by using MZedDB [23], an Aberystwyth University database for accurate mass annotation to 3 (±1) ppm accuracy. The ionisation products of the assigned molecular formulas were first searched against the KEGG compound database specific to *Camellia sinensis* for putative matches. Initial data analysis including classification was performed in R package metabolyseR (0.15.4).

Confirmational analysis (level 1) was performed on a TSQ Quantum Ultra EMR QQQ mass spectrometer (Thermo Fisher Scientific, San Jose, CA, USA) equipped with a heated electrospray ionisation source. Samples were delivered using an Accela UHPLC system (Thermo Fisher Scientific, San Jose, CA, USA) consisting of autosampler, column heater, and quaternary UHPLC-pump. Chromatographic separation was performed on a ZIC-pHILIC (Hydrophilic Interaction Liquid Chromatography polymeric 5 µm, 150 × 4.6 mm) column (Merck, Rahway, NJ, USA) as described [24,25].

### 2.6. Data Analysis

To provide a compositional overview of the samples, consensus structural classifications were compiled for each of the *m*/*z* features that were assigned a molecular formula. To do this, the molecular formulas were first searched against the KEGG compound database. Matching compounds were filtered based on their potential to form the relevant adduct under electrospray ionisation using the MZedDB ionisation rules [23]. Where no compound matches were identified in the KEGG compound database, the molecular formula was instead searched in the PubChem compound database using the same approach. The structural chemical classifications, based on the CHEMONT chemical taxonomy, were retrieved from the ClassyFire database for the matched compounds [26]. For each adduct of each assigned molecular formula, putative structural classifications were assigned to a depth based on a 66% or above consensus between the matched compounds.

Random forest (RF) regression was used to identify *m*/*z* features related to processing steps. RF was selected as the primary machine learning model due to its ability to handle high-dimensional, non-linear data and its robustness to overfitting when applied to relatively small sample sizes. Additionally, its interpretability through variable importance measures allowed us to identify key metabolites contributing to sample differentiation.

Confidence intervals (95%) were reported for key statistical tests to provide a range for expected variability.

*K*-means clustering was performed on the percentage relative abundance of the explanatory features. Functional and structural enrichment analysis was performed on each of the clusters to potentially derive both chemical classes and biological functions related to the cluster trends. Functional enrichment was performed on each of the clusters using the PageRank approach of the FELLA R package [27,28]. Structural enrichment was performed on each of the clusters using over-representation analysis with Fisher’s exact test.

Metabolite identification was achieved at Level 2 (putative annotations based on accurate mass matching) for most features, with selected compounds confirmed at Level 1 using authentic standards. The maximum error was 3 (±1) ppm. Matches were cross-referenced against KEGG and PubChem databases for annotation confidence.

## 3. Results

### 3.1. Structural Composition of the Tea Samples

**Figure 1:**
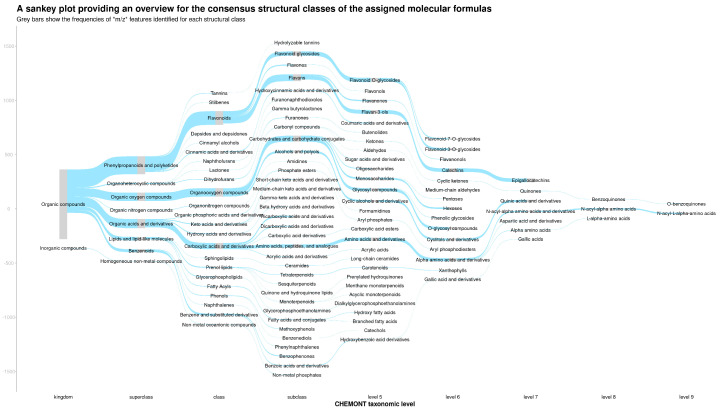
Consensus structural classes of assigned molecular formulas in tea samples. This Sankey plot illustrates the distribution of *m*/*z* features into consensus structural classes based on assigned molecular formulas in tea metabolomic data. Each bar represents the frequency of *m*/*z* features classified under the respective structural class. Grey bars denote the proportional contributions to the overall sample composition. Structural classes were derived using the ClassyFire database based on a 66% consensus threshold.

The ionisation products of the assigned molecular formulas were first searched against the KEGG compound database specific to *Camellia sinensis* for putative matches. Any the structural classifications for any metabolite matches were retrieved from the Classyfire database. For the ionisation products of each assigned molecular formula, a consensus structural classification was assigned based on all the possible database matches using a consensus threshold of 66%. For ionisation products that did not match to the KEGG compound database, these were then matched against the PubChem database and consensus structural classifications assigned accordingly. Figure 1 shows a Sankey plot, which provides an overview of the structural composition of the tea samples. There was a high frequency of *m*/*z* features putatively classified as phenylpropanoids and polyketides, organic oxygen compounds, organic acids and derivatives, lipids and lipid-like molecules, and benzenoids.

### 3.2. Variability Within the Georgian Tea

**Figure 2:**
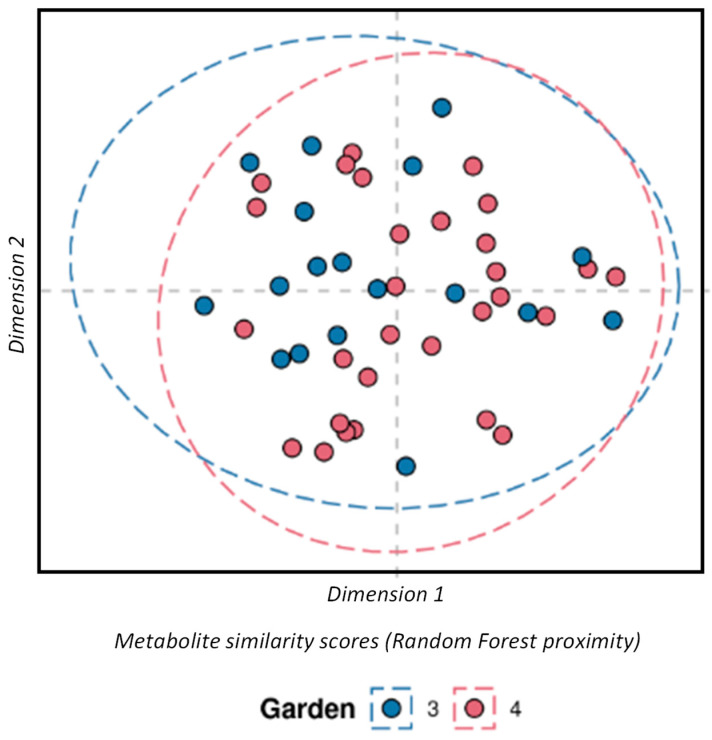
The difference of the Georgian tea variety field samples between the gardens. Multi-dimensional scaling plot of field sample metabolite similarity scores (proximity) from a random forest classification model. Where the eclipse shows the 95% confidence interval for each variety, estimated using the multivariate normal distribution.

**Figure 3:**
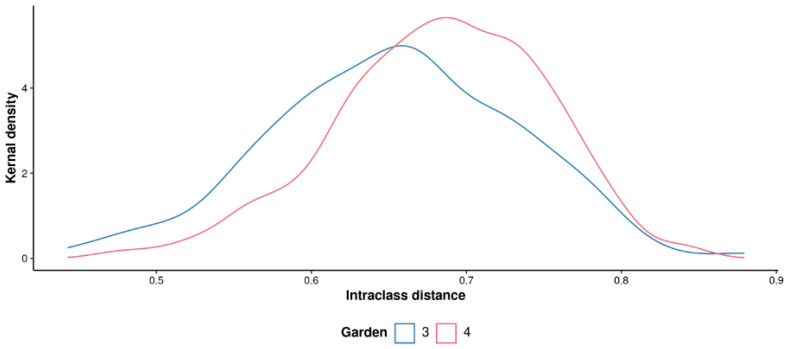
Density distributions of interclass distances between samples of the Georgian tea plants, taken from different gardens. Where interclass distance based on the sample proximity values from a multinomial random classification model, supervised by garden.

RF classification was performed to assess the difference of the Georgian tea plants between the two gardens. As shown in the multi-dimensional scaling plot (MDS) in Figure 2, there was no significant difference found between the plants in these gardens with a margin value of 0.00443 (*p* = 0.572, 3000 permutations). Very similar intraclass variability was also observed in both the gardens as shown by the intraclass distance distributions, where gardens 3 and 4 had median distance values of 0.656 and 0.687, respectively (Figure 3).

### 3.3. Variability Within the Tocklai Tri-Clonal Variants

**Figure 4:**
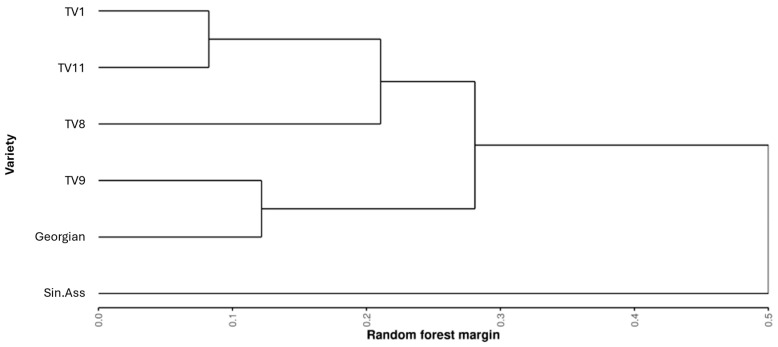
The similarity of tea varieties based on metabolomic fingerprinting. Where similarity based on supervised random forest classification margin values of binary comparisons between each of the tea varieties.

Before assessing the variability within the Tocklai tri-clonal variants, it was first important to establish the relative difference across all the varieties sampled. Pairwise comparisons of RF classification were performed between all the varieties to assess the chemical relatedness between the tea varieties. Figure 4 shows a dendrogram of hierarchical cluster analysis for the resulting RF margin values. All these pairwise comparisons returned significant margin values (*p* < 0.05, 3000 permutations). This shows TV9 was more similar the Georgian varieties than TV8 and TV11, with TV11 showing the greatest similarity to TV1.

**Figure 5:**
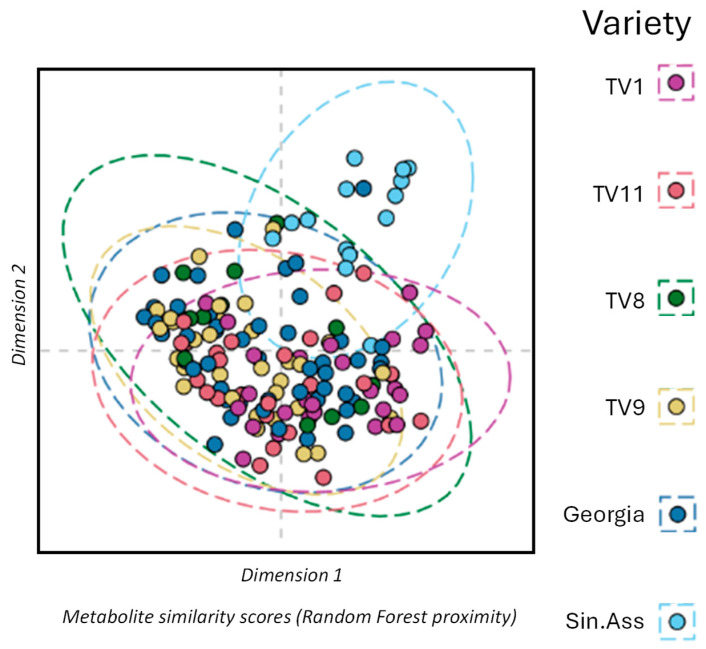
Intraclass variability in tea metabolites across tea varieties. Multi-dimensional scaling plot of field samples similarity (proximity) from a multinominal random forest classification model. Where the eclipse shows the 95% confidence interval for each variety, estimated using the multivariate normal distribution.

To quantify the intraclass variability within the Tocklai tri-clonal variants, all the sampled varieties were compared together using a single multinomial RF classification model. The MDS plot is shown in Figure 5 that is based on the sample proximity values from the RF model. This model gave a weak but significant margin value of 0.0183 (*p* < 0.001, 3000 permutations). The area of the ellipses (95% confidence interval) provides an estimate of the intraclass variability of the collected samples. These ellipses are of similar size across all the varieties, which suggests the observed intraclass variability in leaf composition is similar between the varieties.

### 3.4. Metabolic Changes over the Day in One Variant

**Figure 6:**
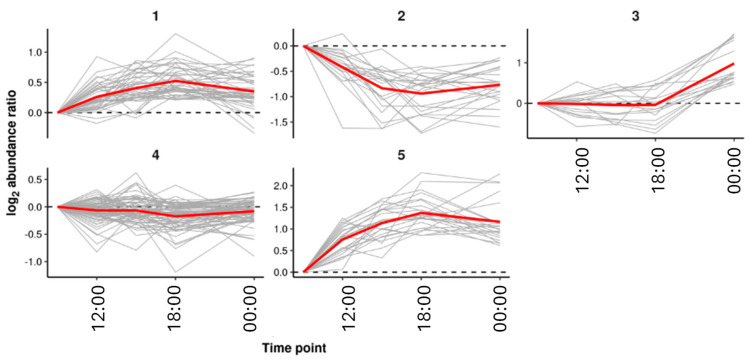
Clusters of explanatory *m*/*z* feature trends across the sampling time points in *Camellia sinensis* var. *assamica* using k-means clustering. Where the abundance radios were calculated using the *m*/*z* feature median values relative to the 09:00 sampling time point. The clusters were identified using *k*-means clustering. Cluster averages are shown in red.

RF regression was used to identify *m*/*z* features related to sampling time point in *C*. *sinensis* var. *assamica*. This yielded a very strong model with a significant R^2^ value of 0.617 (*p* < 0.01, 3000 permutations). There were 174 features (*m*/*z*) found to be explanatory (% increase in mean squared error, *p* < 0.05, 3000 permutations). *k*-means clustering was performed on the log_2_ abundance ratios of the explanatory features taken relative to the first time point (9 a.m.). This grouped the explanatory features into five clusters of features showing similar trends across the sampling time points. The clusters in Figure 6 show the clustering of metabolites based on their temporal abundance patterns. Cluster 1, which includes sucrose, showed a clear diurnal rhythm, indicating its association with daytime photosynthetic activity. Clusters 3 and 5, which include amino acids and flavonoids, may represent biosynthetic pathways that are less directly linked to diurnal cycles and more influenced by other physiological processes.

**Figure 7:**
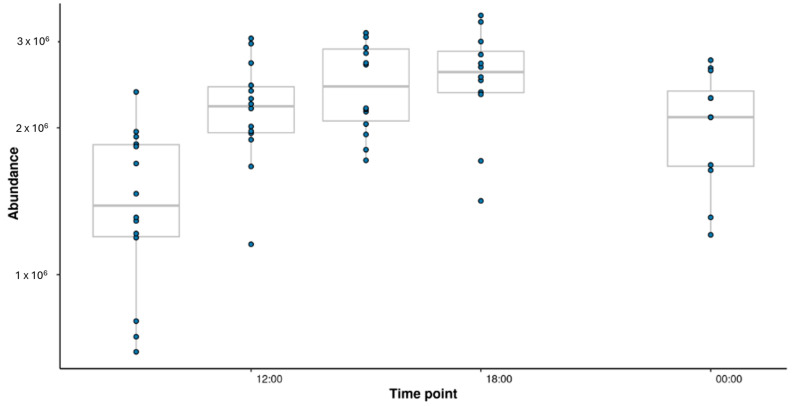
Temporal variation of sucrose abundance in *Camellia sinensis* var. *assamica*. Box plot showing the relative abundance of sucrose [M-H]^−^ across different time points in *Camellia sinensis* var. *assamica*. Data were normalised and log2 transformed. The box represents the interquartile range (IQR), with the median indicated by a horizontal line. Whiskers denote 1.5 times the IQR and dots represent outliers.

Functional and structural enrichment analysis was performed on each of the clusters to potentially derive both chemical classes and biological functions related to the cluster trends. This identified significant metabolic pathways (Table 1). Structural enrichment was performed with over-representation analysis using Fisher’s exact test (Table 2). Both the structural subclass ‘Carbohydrates and carbohydrate conjugates’ and the metabolic pathway ‘Starch and sucrose metabolism’ were found to be significant for cluster 1, where the abundance of these features increase throughout daylight hours, peaking at 18:00 and then began to decrease. Figure 7 highlights the diurnal variation in sucrose abundance in *Camellia sinensis* var. *assamica*. The observed increase during daylight hours followed by a decline at night suggests a link between sucrose metabolism and photosynthetic activity. This aligns with the identification of starch and sucrose metabolism as a key pathway (Table 1, cluster 1). Sucrose was confirmed to Level 1 with standards using HILIC.

## 4. Discussion and Conclusions

Studies have demonstrated the effectiveness of metabolomics in distinguishing tea varieties and assessing quality markers [13,14,15]. Our study adds to this research and provides a detailed exploration of the chemical diversity and variability in tea samples from the Dartmoor Estate plantation in Devon, UK, highlighting the influence of cultivar, geographical location, and temporal factors. The findings demonstrate that while no significant differences were observed between Georgian garden locations, substantial chemical variability exists within tri-clonal Tocklai tea variants. Specifically, TV9 showed a greater similarity to Georgian cultivars than TV8 and TV11, with TV11 most closely related to TV01. These results underscore the role of genetic variation in influencing tea’s chemical composition and the potential for tailoring cultivation strategies to optimise specific traits. While statistical significance was achieved across most analyses, it is essential to interpret these results in the context of effect sizes and model power. However, future studies with larger sample sizes are warranted to confirm these findings and further enhance model robustness.

Temporal analyses of *Camellia sinensis* var. *assamica* revealed dynamic metabolic changes over the course of the day, particularly in starch and sucrose metabolism (cluster 1). The abundance of related metabolites increased during daylight hours before declining at night, demonstrating the importance of diurnal rhythms in influencing tea leaf composition. This highlights the potential for targeted harvesting schedules to maximise desired chemical profiles. This temporal regulation may influence not only the chemical composition of tea but also its flavour profile and quality. In another published study, High Performance Liquid Chromatography showed sucrose concentrations in the third and fourth tea leaves were significantly higher (*p* ≤ 0.05) than that of the bud, first, and second leaves. However, there was no significant effect of time of plucking affecting sucrose, concentrations in fresh leaves [29], unlike our results. On the other hand, it has been demonstrated the significant impact of seasonal changes on tea metabolite profiles, highlighting the intricate relationship between gene expression and metabolite biosynthesis [30]. Our observed chemical variability aligns with studies from traditional tea-growing regions. Significant climatic impacts on tea metabolite profiles in Assam have been reported [7], while seasonal changes were found in the amino acid concentrations in tea [31]. The enrichment of flavonoids in cluster 4 suggests a potential link to stress responses or secondary metabolism, which may be less directly tied to diurnal rhythms. These patterns align with known metabolic adaptations in plants to optimise growth and defence under variable environmental conditions.

The application of non-targeted metabolomics, FIE-MS, uncovered subtle compositional differences and identities of key metabolic pathways. By integrating metabolomics with functional and structural enrichment analyses (machine learning), this research advances our understanding of the complex interplay of environmental, genetic, and temporal factors in tea cultivation.

The review by [32] highlights the role of metabolomics in tea cultivation and processing, emphasising its ability to provide comprehensive chemical profiles that inform breeding strategies, cultivation practices, and processing methods. Our insights contribute to this broader knowledge of tea science, emphasising the importance of precision in agricultural and processing practices. They also offer valuable guidance for optimising tea production, enhancing its sensory qualities and bioactive potential, and preserving its cultural and economic significance in the global tea industry. While our study primarily focused on the metabolomic profiling of fresh tea leaves, it is well-documented that drying, fermentation, oxidation, and heat treatment significantly influence the chemical composition and sensory attributes of tea [33]. We have recently published insightful data on the metabolic pathways influencing tea processing, underscoring the importance of these variables in determining the final chemical profile and organoleptic characteristics of tea products [34].

The preparation of tea leaves for analysis traditionally considers developmental phases, from the collection of tender tips to fully mature leaves, which are known to influence chemical profiles and tea quality. While our study focused on standardised sampling of leaves across different cultivars and locations to minimise variability due to developmental stages, future research could include a stratified approach to analyse tips, flushes, and mature leaves separately to better capture the dynamic metabolomic changes during leaf development. Additionally, environmental factors such as soil composition, sunlight exposure, and altitude, which were monitored in this study, could be explored to see their impact on metabolomic profiles and tea quality.

A direct comparison of the metabolomic profiles of UK-grown tea with those from traditional tea-growing regions such as Georgia, India, and China would provide valuable insights into the influence of geography and cultivation practices on tea chemistry. Such comparative studies could highlight similarities and differences in secondary metabolite composition, contributing to an understanding of how environmental factors affect tea quality.

This research contributes to the growing field of metabolomics by providing a comprehensive chemical profile of tea grown in a non-traditional region. The integration of metabolomics with machine learning, specifically RF classification, enabled us to identify significant chemical variability across cultivars and time points. These findings offer new insights into the adaptability of tea plants and their potential for cultivation in emerging regions, contributing to global food security and agricultural diversification. This approach highlights the potential of machine learning tools in metabolomics for uncovering complex patterns in high-dimensional data. The approach used in this paper is robust for generating hypotheses and identifying broad trends; however, annotations based solely on molecular formulas remain tentative until confirmed by standards.

While this study provides valuable insights, it has certain limitations. The sampling was confined to a single growing season and location, limiting the ability to generalise findings across multiple climates or years. Additionally, the metabolomic analysis was based on fresh leaves and the effects of processing methods, such as drying and fermentation, were not explored. Future research should include multi-seasonal sampling, comparisons with traditional tea-growing regions, and analysis of processed tea to provide a more comprehensive understanding of tea chemistry.

To ensure robust biomarker discovery in metabolomics, replication across different harvest years and environmental conditions is critical. Variability in metabolomic profiles can arise due to biological factors such as plant age and environmental factors like soil composition, weather conditions, and harvest timing. High replication numbers, typically ranging between 9 and 24 replicates, are often necessary to increase confidence in observed trends while accounting for such variability. However, increasing replication beyond this range may inadvertently decrease the standard error to levels that favour outcomes arising by chance, thus underscoring the importance of balancing replication with experimental design rigor.

In this study, the replication numbers vary significantly, ranging from 3 at certain time points to 48 for specific varieties (e.g., Georgian). This variability reflects the exploratory nature of the research, which aimed to generate hypotheses regarding the chemical diversity of tea grown in a non-traditional region. While such variability is inherent to early-stage studies, it introduces potential biases that should be addressed in future investigations. For instance, future experiments could include replication across multiple harvest years under consistent conditions to verify that the observed patterns are not due to temporal or environmental chance events.

These considerations align with our objective to refine this metabolomics approach for hypothesis generation rather than clinical or diagnostic applications [35,36]. To address these potential biases and enhance robustness, future studies should prioritize controlled experimental designs with balanced replication and incorporate additional environmental and biological parameters to validate the findings presented here.

Future studies should aim to further investigate the relationship between chemical composition and sensory attributes, as well as the role of microbial and environmental interactions in influencing tea quality.

## Figures and Tables

**Table 1:** Functional enrichment analysis was performed on each of the clusters to potentially derive both biochemical pathways and biological functions related to the cluster trends.

Cluster	KEGG id	KEGG Name	*p*-Score
1	csin00020	Citrate cycle (TCA cycle)	0.0000
1	csin00040	Pentose and glucuronate interconversions	0.0000
1	csin00053	Ascorbate and aldarate metabolism	0.0000
1	csin00500	Starch and sucrose metabolism	0.0000
1	csin00600	Sphingolipid metabolism	0.0000
1	csin00603	Glycosphingolipid biosynthesis	0.0000
1	csin00660	C5-Branched dibasic acid metabolism	0.0000
1	csin00760	Nicotinate and nicotinamide metabolism	0.0000
1	csin02010	ABC transporters	0.0000
1	csin04016	MAPK signaling pathway	0.0000
3	csin00030	Pentose phosphate pathway	0.0472
3	csin00040	Pentose and glucuronate interconversions	0.0054
3	csin00240	Pyrimidine metabolism	0.0110
3	csin00250	Alanine, aspartate and glutamate metabolism	0.0000
3	csin00280	Valine, leucine and isoleucine degradation	0.0000
3	csin00330	Arginine and proline metabolism	0.0001
3	csin00410	beta-Alanine metabolism	0.0001
3	csin00470	D-Amino acid metabolism	0.0408
3	csin00511	Other glycan degradation	0.0394
3	csin00561	Glycerolipid metabolism	0.0000
3	csin00620	Pyruvate metabolism	0.0000
3	csin00670	One carbon pool by folate	0.0000
3	csin00740	Riboflavin metabolism	0.0000
3	csin00970	Aminoacyl-tRNA biosynthesis	0.0000
3	csin01200	Carbon metabolism	0.0000
3	csin04148	Efferocytosis	0.0000
4	csin00941	Flavonoid biosynthesis	0.0000
4	csin00999	Biosynthesis of various plant secondary metab...	0.0001
5	csin00030	Pentose phosphate pathway	0.0000
5	csin00040	Pentose and glucuronate interconversions	0.0000
5	csin00053	Ascorbate and aldarate metabolism	0.0000
5	csin00250	Alanine, aspartate and glutamate metabolism	0.0000
5	csin00270	Cysteine and methionine metabolism	0.0000
5	csin00290	Valine, leucine and isoleucine biosynthesis	0.0000
5	csin00330	Arginine and proline metabolism	0.0042
5	csin00750	Vitamin B6 metabolism	0.0000
5	csin01230	Biosynthesis of amino acids	0.0019
5	csin04016	MAPK signaling pathway	0.0000

**Table 2:** Structural enrichment analysis was performed on each of the clusters to potentially derive both chemical classes and biological functions related to the cluster trends.

Cluster	Classification	*p*-Value	Adjusted *p*-Value
1	Organic compounds	0.000	0.002
1	Organic acids and derivatives	0.002	0.052
1	O-glycosyl compounds	0.003	0.056
1	Carbohydrates and carbohydrate conjugates	0.003	0.068
1	Keto acids and derivatives	0.003	0.072
1	Glycosyl compounds	0.004	0.078
1	Organooxygen compounds	0.010	0.224
1	Organic oxygen compounds	0.010	0.224
1	Tricarboxylic acids and derivatives	0.010	0.225
1	Medium-chain keto acids and derivatives	0.011	0.246
1	Pentoses	0.022	0.490
1	Gamma-keto acids and derivatives	0.022	0.490
1	Carboxylic acids and derivatives	0.025	0.554
1	Alpha amino acids	0.044	0.969
2	Cyclic alcohols and derivatives	0.005	0.126
2	Cyclitols and derivatives	0.005	0.126
2	Quinic acids and derivatives	0.005	0.126
2	Alcohols and polyols	0.005	0.126
2	Organic compounds	0.006	0.154
2	Organooxygen compounds	0.009	0.227
2	Organic oxygen compounds	0.009	0.227
2	Sphingolipids	0.011	0.286
2	Long-chain ceramides	0.011	0.286
2	Ceramides	0.011	0.286
2	Non-metal oxoanionic compounds	0.017	0.427
2	Inorganic compounds	0.017	0.427
2	Non-metal phosphates	0.017	0.427
2	Homogeneous non-metal compounds	0.017	0.427
2	Cinnamic acids and derivatives	0.040	0.986
2	Coumaric acids and derivatives	0.040	0.986
2	Hydroxycinnamic acids and derivatives	0.040	0.986
3	Hydroxy acids and derivatives	0.001	0.005
3	Organic acids and derivatives	0.006	0.057
3	Aspartic acid and derivatives	0.013	0.132
4	No database hits	0.001	0.009
5	Amino acids and derivatives	0.014	0.206
5	Alpha amino acids and derivatives	0.014	0.206
5	Amino acids, peptides, and analogues	0.014	0.206
5	Hydroxy fatty acids	0.019	0.280
5	Aspartic acid and derivatives	0.019	0.280
5	Alpha amino acids	0.025	0.372
5	Fatty Acyls	0.031	0.463
5	Fatty acids and conjugates	0.031	0.463
5	Carboxylic acids and derivatives	0.049	0.727

## Data Availability

The metabolomics and metadata reported in this paper are available on request.

## Supplementary Materials

The following supporting information can be downloaded at: https://www.mdpi.com/article/10.3390/metabo15010052/s1, Figure S1. Randomised sampling map was computer-generated before the investigation to account for batches, location (garden/plot), and time taken. Figure S2. Pre-treatment parameters.

## Abbreviations

The following abbreviations are used in this manuscript. GRGN (Georgian N2 cultivar), TV 08, 09, 11, and 01 (*Camellia sinensis* L. Tocklai Tri-clonal Variant), Sin.Ass = Camellia assamica Masters; syn C. sinensis var assamica variety), (FIE-MS) Flow Infusion Electrospray Ionisation Mass Spectrometry, (HILIC) Hydrophilic interaction liquid chromatography, (RF) random forest, (MDS) multi-dimensional scaling plot.
