# Correction: Lloyd et al. Chemical Diversity of UK-Grown Tea Explored Using Metabolomics and Machine Learning. *Metabolites* 2025, *15*, 52

**DOI:** 10.3390/metabo15080538

**Published:** 2025-08-08

**Authors:** Amanda J. Lloyd, Alina Warren-Walker, Jasen Finch, Jo Harper, Kathryn Bennet, Alison Watson, Laura Lyons, Pilar Martinez Martin, Thomas Wilson, Manfred Beckmann

**Affiliations:** 1 Department of Life Sciences, Aberystwyth University, Aberystwyth SY23 3DA, UK; jsf9@aber.ac.uk (J.F.); aan@aber.ac.uk (A.W.); lal8@aber.ac.uk (L.L.); mam167@aber.ac.uk (P.M.M.); tpw2@aber.ac.uk (T.W.); meb@aber.ac.uk (M.B.); 2 Dartmoor Estate Tea, Furzeleigh Farm, Ashburton, Newton Abbot TQ13 7JL, UK; jo.harper4@gmail.com (J.H.); dartmoorestatetea@gmail.com (K.B.)

## Text Correction

There was a provenance issue with the seed varieties in the original publication [1].

A correction has been made to Materials and Methods, 2.1. Tea Plants:

Six tea varieties were available at Dartmoor Estate Tea, UK, in September: GRGN (ex-Soviet Georgia commercial seed; 535 plants available and 48 plants were randomly sampled between two gardens); TV 08, 09, 11, and 01 (*Camellia sinensis* originally sourced from the Tocklai biclonal seed series TS506, TS557, TS589, and Tocklai Darjeeling commercial seed variety BB668, respectively; 860 available plants and 128 plants were randomly sampled between two gardens); and Sin.Ass, which was Tocklai Biclonal TS378 (1560 available plants in one garden; 64 plants were randomly sampled). A randomised sampling map was computer-generated before the investigation to account for batches, location (garden/plot), and time taken (Supplementary Data, Figure S1). All seeds were supplied by Teacraft Ltd., UK. These seed-derived varieties were previously misattributed as Tocklai TV vegetatively propagated clones (TV8, TV9, TV11, and TV1) in the earlier manuscript. However, we now clarify that these are not vegetatively propagated clones but seed-derived lines, and the Teacraft Ltd. codes (TCL01, TCL08, TCL09, TCL11, and TEV01, respectively) should be used throughout this revised version to reflect their correct provenance (TS378, TS506, TS557, TS589, and BB668, respectively). The Georgian seed (GRGN) was collected from abandoned tea estates in western Georgia, representing a mixed genetic background rather than a specific cultivar [20].

The authors state that the scientific conclusions are unaffected. This correction was approved by the Academic Editor. The original publication has also been updated.
