# Supplementary material for: Chemical Diversity of UK-Grown Tea Explored Using Metabolomics and Machine Learning
Source: Metabolites. 2025 Jan 15;15(1):52. doi: 10.3390/metabo15010052 (PMC11767213; doi:10.3390/metabo15010052)
Supplement: Supplementary file 1 [file metabolites-15-00052-s001.zip › metabolites-3433008-supplementary.pdf]

Figure S1. A randomised sampling map was computer-generated before the investigation to account for batches, location (garden/plot) and time taken

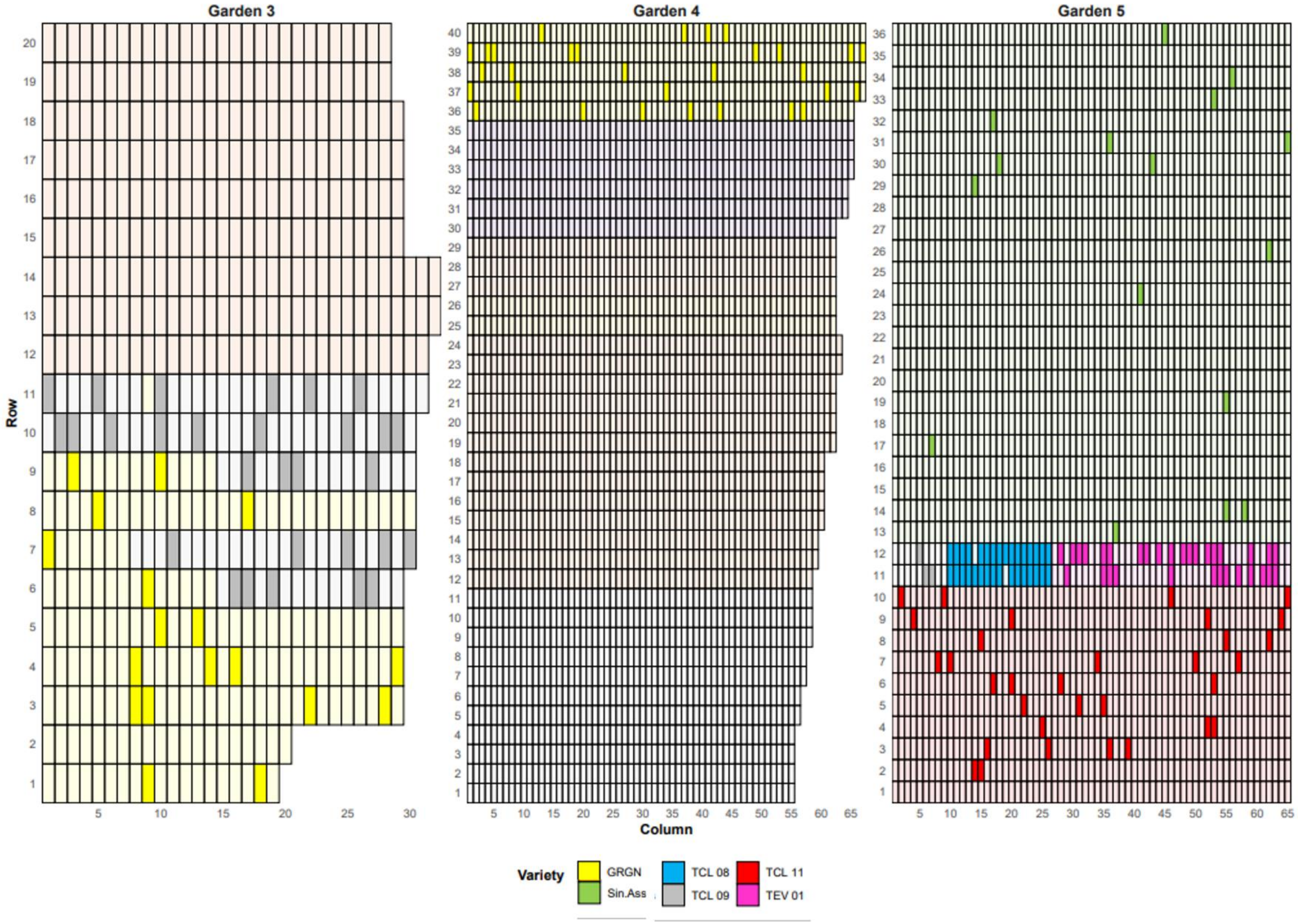

Figure S2 Pre-Treatment parameters

```
## pre-treatment
##  QC
##     occupancyFilter
##        cls = class
##        QCidx = QC
##        occupancy = 2/3
##     impute
##        cls = class
##        QCidx = QC
##        occupancy = 2/3
##        parallel = variables
##        seed = 1234
##     RSDfilter
##        cls = class
##        QCidx = QC
##        RSDthresh = 50
##     removeQC
##        cls = class
##        QCidx = QC
##  occupancyFilter
##     maximum
##        cls = class
##        occupancy = 2/3
##  impute
##     class
##        cls = class
##        occupancy = 2/3
##        seed = 1234
##  transform
##     TICnorm
```

```
##         refactor = TRUE
```
